# Cardiac Medication Prescription Pattern and Association With Transplant-free Survival in the Adult Fontan Population

**DOI:** 10.1016/j.jacadv.2025.102070

**Published:** 2025-08-20

**Authors:** Andrew M. Freddo, Molly Eron, Antara Mondal, Alexis Z. Tomlinson, Srinivas Denduluri, Sara Partington, Emily Ruckdeschel, Allison L. Tsao, Constantine D. Mavroudis, Muhammad Nuri, Stephanie Fuller, Juan M. Ortega-Legaspi, Yuli Y. Kim, Sumeet Vaikunth

**Affiliations:** a Division of Cardiology, Department of Pediatrics, Children's Hospital of Philadelphia, Philadelphia, Pennsylvania, USA; b Division of Cardiovascular Medicine, Department of Medicine, Hospital of the University of Pennsylvania, Philadelphia, Pennsylvania, USA

**Keywords:** adult congenital heart disease, Fontan, outcomes, pediatric cardiology, single ventricle

## Abstract

**Background:**

There are limited data on the cardiac medications taken by patients with Fontan circulation in adult congenital heart disease (ACHD) clinics.

**Objectives:**

The objectives of the study were to describe the type and frequency of cardiac medications prescribed to adults with Fontan circulation and assess for association between medication prescriptions/burden and transplant-free survival.

**Methods:**

A retrospective cohort study of adults with Fontan circulation followed in an outpatient ACHD clinic between 2009 and 2023 was performed. Demographics, clinical variables, and all prescribed outpatient cardiac medications were abstracted from the electronic medical record. The outcome of interest was transplant-free survival. Cox proportional hazards analysis was performed to determine the association between the medication type/burden and transplant-free survival.

**Results:**

There were 429 patients (55.2% male, 72.5% White, and 49.7% single left ventricle morphology) with median age 24 years (IQR: 22-29) at time of first clinic visit. The most prescribed medication classes were antiplatelet agents (74.6%) followed by angiotensin converting enzyme inhibitors or angiotensin receptor blockers (59.0%), anticoagulants (44.1%), loop diuretics (39.6%), metoprolol (32.2%), mineralocorticoid receptor antagonists (31.5%), antiarrhythmics (27.5%), and pulmonary vasodilators (16.1%). Being prescribed loop diuretic agents was associated with worse transplant-free survival (HR: 9.49, *P* < 0.001).

**Conclusions:**

We report one of the first large-scale studies of prescription patterns in adult patients with Fontan circulation in a single ACHD clinic. We demonstrate an inverse association between loop diuretic prescriptions and transplant-free survival.

Since the advent of the Fontan operation in the late 1960s for tricuspid atresia^1^ and subsequent modifications thereafter, an increasing number of patients surviving into adulthood are seeking adult congenital heart disease (ACHD) care. Unlike acquired heart disease, for which practice guidelines on medical therapy exist based on high-level evidence, there are limited data-driven guidelines for medical therapy in ACHD. Without these guidelines, it is likely that prescription patterns vary. Previous work has shown large variation in prescription practices in pediatric age patients with Fontan circulation,^2^ but few studies describe medications in the adult. There also are limitations in the ability to prospectively follow patient response to therapy given the extended timeline for adverse events to occur, heterogeneity of the population, and variations between centers.^3^ Limited placebo-controlled clinical trials, such as the FUEL (Fontan Udenafil Exercise Longitudinal) and RUBATO trials, have attempted to evaluate the effect of medications on surrogate outcomes, such as measures of oxygen consumption from cardiopulmonary exercise testing, although with mixed results.^4^^,^^5^ Medications prescribed can reflect both comorbid conditions as well as illness severity, which predict long-term clinical outcomes. Here we describe medications prescribed to patients with Fontan circulation at our ACHD center and explore the relationship between medication prescriptions and adverse outcomes.

## Methods

### Data source and study design

A retrospective cohort was identified by searching the electronic medical record (EMR) for adults ≥18 years of age using International Classification of Diseases-Ninth Revision and International Classification of Diseases-10th Revision codes indicating Fontan circulation (Supplemental Table 1) who had at least one office visit encounter in the outpatient ACHD clinic between January 1, 2009, and December 31, 2023. Additional clinical data abstracted from the EMR included demographics, cardiac diagnosis, details of Fontan operation, and cardiac and extracardiac comorbidities. The outcome of interest was transplant-free survival. For cardiac and extracardiac comorbidities, these were noted if listed in the patient's problem list or provider documentation. For arrhythmia, this included bradyarrhythmias (including sinus node dysfunction and high-degree atrioventricular block) and tachyarrhythmias (including atrial and ventricular arrhythmias). This included findings on Holter monitor, when available, and excluded isolated asymptomatic premature atrial or ventricular contractions. For cirrhosis, this included either clinical findings of cirrhosis in ACHD or hepatology documentation and/or imaging findings consistent with cirrhosis. Follow-up was defined as the time from the first clinic visit until either the last confirmed contact in the EMR (up to and including January 31, 2024) or the date of heart transplant or death. This study was approved by the University of Pennsylvania IRB 827550.

### Medications

For each patient, medication history (including medication names, start dates, and end dates) was automatically collected. Medications were filtered, and the following categories of medications were removed: noncardiac medications, inpatient medications, and pulmonary vasodilators with an indication for erectile dysfunction. Start and end dates obtained in the initial data collection were verified by chart review, and medications that had a start and end date ≤10 days apart were removed. Medications were collated by generic name and grouped into medication classes and superclasses based on clinical indication. Metoprolol succinate and tartrate were separated from other beta blockers because they could be prescribed for multiple indications (eg, heart failure or arrhythmia). Calcium channel blockers and other beta blockers were separated based on typical indication, antiarrhythmic or not. These classes and superclasses are summarized in Supplemental Table 2. If a patient had a medication class prescribed at any point during their follow-up, they were included in that medication class group. For each patient, total medication burden was defined as the number of superclasses prescribed (as defined in Supplemental Table 2).

### Data analysis

Descriptive statistics were reported as frequencies and percentages for categorical variables, and median and IQR for continuous variables. Cox regression was performed to determine if various medication classes were associated with transplant-free survival. Transplant-free survival was defined as no heart transplant or death before the patient's last contact date with our health system. Patients were followed from their first encounter in the ACHD clinic as reported in the EMR to the date of heart transplant or death, whichever came first. Patients who did not experience heart transplant or death were censored at their last contact date with our health system.

#### Medications prescribed

The independent association between each medication class and transplant-free survival from the first ACHD encounter was initially assessed using univariable Cox proportional hazard models. Medication classes with significant univariable relationships were then analyzed jointly in a multivariable Cox proportional hazard model.

Sensitivity analyses were also performed to assess the potential influence of several confounders on the effect of medication classes that had significant associations in the multivariable model. Confounders were identified as follows: for each medication class, univariable logistic regression was performed to identify whether each potential confounder was significantly associated with the prescription of the medication class. Confounders with significant univariable associations and determined to be clinically relevant to prescription of the medication class were then included in a multivariable logistic model. Significant confounders in the multivariable logistic model were then included in a final multivariable Cox model that included both confounders and medication classes.

#### Medication burden

The independent association between medication burden and transplant-free survival was assessed using univariable Cox regression. A second sensitivity analysis was performed as described above to assess the potential influence of confounders on medication burden and transplant-free survival.

Statistical analysis was performed using STATA (version 18, StataCorp). The proportional hazards assumption was assessed for all Cox models. Significance for all statistical tests was defined as *P* < 0.05.

## Results

### Patient characteristics

The underlying characteristics of the studied cohort is summarized in Table 1 and comorbidities summarized in Table 2 and Supplemental Table 3. Of 429 patients studied, 237 (55.2%) were male and 311 (72.5%) were White. The median age at the patient's first ACHD clinic visit was 24 years old, with an IQR of 22 to 29 years old. Median follow-up time was 3.97 years, with an IQR of 1.58 to 7.14 years. Just under half of patients had single left ventricle morphology (213, 49.7%) and the most common primary diagnosis was tricuspid atresia (114, 26.5%). The most common Fontan type was a lateral tunnel (253, 59.1%).

**Table 1:** Patient Characteristics of Cohort (N = 429)

Male	237 (55.2)
Age at first ACHD visit, y	24 [22-29]
Follow-up time, y	3.97 [1.58-7.14]
Race	
Asian	11 (2.6)
Black	45 (10.5)
White	311 (72.5)
Other	18 (4.2)
Unknown	44 (10.3)
Hispanic/Latino ethnicity	20 (4.7)
Primary cardiac diagnosis	
Tricuspid atresia	114 (26.5)
HLHS	109 (25.4)
DORV	59 (13.8)
DILV	58 (13.5)
Unbalanced atrioventricular canal	42 (9.8)
PA/IVS	25 (5.8)
Other	22 (5.1)
Ventricular morphology	
Single left ventricle	213 (49.7)
Single right ventricle	187 (43.6)
Mixed ventricular morphology	29 (6.8)
Other characteristics	
Bilateral SVC	70 (16.3)
Heterotaxy	52 (12.1)
Fontan type	
Lateral tunnel	253 (59.1)
Extracardiac	87 (20.3)
Aortopulmonary	60 (14.0)
Other	28 (6.5)
Fenestrated	243 (56.6)
Age at Fontan, months	29.71 [20.89-52.90]
Fontan revision	56 (13.1)
Fontan completion date	
1976-1990	114 (26.6)
1991-2000	231 (54.0)
2001-2014	83 (19.4)

Values are n (%) or median [IQR].

ACHD = adult congenital heart disease; DILV = double-inlet left ventricle; DORV = double-outlet right ventricle; HLHS = hypoplastic left heart syndrome; PA/IVS = pulmonary atresia with intact ventricular septum; SVC = superior vena cava.

**Table 2:** Cardiac and Fontan-Specific Comorbidities

Cardiovascular comorbidities	
Arrhythmia	255 (59.4)
Pacemaker/implantable cardioverter Defibrillator	107 (24.9)
Systolic and/or diastolic heart failure	93 (21.7)
Stroke/transient ischemic attack	85 (19.8)
Hypertension	46 (10.7)
Valve repair	19 (4.4)
Valve replacement	15 (3.5)
Pulmonary hypertension	15 (3.5)
Endocarditis	12 (2.8)
Hyperlipidemia	11 (2.6)
Myocardial infarction	4 (0.9)
Coronary artery disease	1 (0.2)
No cardiovascular comorbidities	112 (26.1)
Fontan comorbidities	
Cyanosis (SpO2 <90%)	111 (25.9)
Fontan thrombus	44 (10.3)
Pulmonary arteriovenous malformations	35 (8.2)
Protein losing enteropathy	30 (7.0)
Plastic bronchitis	1 (0.2)
No fontan comorbidities	264 (61.5)

Values are n (%).

### Medications prescribed

The medications prescribed to patients in the cohort are summarized in Table 3. The most prescribed classes were antiplatelets (320 patients, 74.6% of the total cohort), angiotensin-converting enzyme inhibitors (ACEi) (224, 52.2%), loop diuretics (170, 39.6%), metoprolol (138, 32.2%), and mineralocorticoid receptor antagonists (MRA) (135, 31.5%). Certain classes were combined into superclasses of medications: ACEi/angiotensin receptor blocker (ARB)/angiotensin receptor/neprilysin inhibitor (253, 59.0%), anticoagulants, including direct oral anticoagulants (DOAC), heparin analogs, and warfarin (189, 44.1%), diuretic agents, including both loop and thiazide diuretic agents (175, 40.8%), and antiarrhythmics (118, 27.5%).

**Table 3:** Cardiac Medications Prescribed

Medication Superclass	N (%)	Medication Class	N (%)	Medication (Generic)	N (%)
ACEI/ARB/ARNI	253 (59.0)	ACEi	224 (52.2)	Captopril	4 (0.9)
				Enalapril	131 (30.5)
				Lisinopril	113 (26.3)
				Quinapril	1 (0.2)
				Ramipril	4 (0.9)
		ARB	46 (10.7)	Irbesartan	2 (0.5)
				Losartan	42 (9.8)
				Olmesartan	1 (0.2)
				Telmisartan	1 (0.2)
				Valsartan	3 (0.7)
		ARNI	4 (0.9)	Sacubitril-valsartan	4 (0.9)
Anticoagulants	189 (44.1)	DOAC	85 (19.8)	Apixaban	55 (12.8)
				Dabigatran	6 (1.4)
				Edoxaban	1 (0.2)
				Rivaroxaban	36 (8.4)
		Heparin analog	49 (11.4)	Dalteparin	1 (0.2)
				Enoxaparin	48 (11.2)
				Heparin	2 (0.5)
		Warfarin	119 (27.7)	Warfarin	119 (27.7)
Diuretics	175 (40.8)	Loop diuretic	170 (39.6)	Bumetanide	36 (8.4)
				Furosemide	143 (33.3)
				Torsemide	44 (10.3)
		Thiazide diuretic	44 (10.3)	Chlorothiazide	7 (1.6)
				Chlorthalidone	4 (0.9)
				Hctz	11 (2.6)
				Indapamide	1 (0.2)
				Metolazone	28 (6.5)
		Other diuretic	1 (0.2)	Amiloride	1 (0.2)
Antiarrhythmics	118 (27.5)	Other antiarrhythmic	84 (19.6)	Amiodarone	30 (7.0)
				Dofetilide	29 (6.8)
				Flecainide	3 (0.7)
				Mexiletine	3 (0.7)
				Propafenone	2 (0.5)
				Sotalol	37 (8.6)
		Antiarrhythmic beta blocker	44 (10.3)	Atenolol	24 (5.6)
				Nadolol	16 (3.7)
				Propranolol	5 (1.2)
		Antiarrhythmic calcium channel blocker	16 (3.7)	Diltiazem	14 (3.3)
				Verapamil	3 (0.7)
Antiplatelets			320 (74.6)	Aspirin	319 (74.3)
				Clopidogrel	6 (1.4)
				Ticagrelor	1 (0.2)
Metoprolol			138 (32.2)	Metoprolol succinate	120 (28.0)
				Metoprolol tartrate	55 (12.8)
Mineralocorticoid receptor antagonists			135 (31.5)	Eplerenone	24 (5.6)
				Spironolactone	122 (28.4)
Digoxin			87 (20.3)	Digoxin	87 (20.3)
Pulmonary vasodilators			69 (16.1)	Bosentan	2 (0.5)
				Macitentan	2 (0.5)
				Sildenafil	53 (12.4)
				Tadalafil	18 (4.2)
				Treprostinil	1 (0.2)
				Udenafil	1 (0.2)
Other beta blockers			45 (10.5)	Bisoprolol	2 (0.5)
				Carvedilol	37 (8.6)
				Labetalol	6 (1.4)
				Pindolol	1 (0.2)
Statins			17 (4.0)	Atorvastatin	13 (3.0)
				Lovastatin	2 (0.5)
				Rosuvastatin	2 (0.5)
				Simvastatin	3 (0.7)
GLP1			10 (2.3)	Dulaglutide	2 (0.5)
				Liraglutide	3 (0.7)
				Semaglutide	8 (1.9)
Other calcium channel blockers			9 (2.1)	Amlodipine	9 (2.1)
				Felodipine	1 (0.2)
SGLT2i			8 (1.9)	Dapagliflozin	3 (0.7)
				Empagliflozin	5 (1.2)

Note that some patients were prescribed multiple medications within a class or superclass, and thus the total number of generic prescriptions does not equal the number of patients included in that class.

ACEI = angiotensin converting enzyme inhibitors, ARB = angiotensin receptor blockers, ARNI = angiotensin receptor/neprilysin inhibitor, DOAC = direct oral anticoagulant, GLP1 = glucagon-like peptide-1 agonist, SGLT2i = sodium-glucose cotransporter-2 inhibitors.

### Medications and outcomes

The outcome of interest, death or transplant, occurred in 55 patients (12.8%). Overall transplant-free survival 5, 10, and 15 years after establishing ACHD care was 88% (84%-91%), 80% (73%-85%), and 66% (52%-76%), respectively.

Univariable analysis showed increased hazard of death or transplant after the first ACHD encounter in patients prescribed loop diuretic agents (HR: 25.81; *P* < 0.001), MRA (HR: 5.87; *P* < 0.001), pulmonary vasodilators (HR: 3.31; *P* < 0.001), anticoagulants (HR: 2.63; *P* = 0.001), antiarrhythmics (HR: 2.36; *P* = 0.002), digoxin (HR: 2.30; *P* = 0.002), and nonantiarrhythmic beta blockers (HR: 2.21; *P* = 0.013). DOAC prescriptions trended toward decreased hazard of death or transplant (HR: 0.51; *P* = 0.095) (Table 4).

**Table 4:** Association Between Medication Class Prescribed and Transplant-free Survival From First ACHD Encounter

Medication Class	HR	95% CI	*P* Value
Univariable regressions			
Superclasses			
MRA	5.87	3.24-10.62	<0.001
Pulmonary vasodilator	3.31	1.92-5.71	<0.001
Anticoagulant	2.63	1.45-4.77	0.001
Antiarrhythmic	2.36	1.38-4.02	0.002
Digoxin	2.30	1.34-3.94	0.002
Other beta blockers	2.21	1.19-4.12	0.013
Metoprolol	1.43	0.84-2.43	0.192
ACEi/ARB/ARNI	0.87	0.51-1.50	0.617
Classes			
Loop diuretic	25.81	8.06-82.66	<0.001
Warfarin	3.41	1.96-5.92	<0.001
Other antiarrhythmic	3.03	1.78-5.16	<0.001
Antiarrhythmic beta blockers	0.98	0.44-2.18	0.967
ACEi	0.85	0.50-1.44	0.547
ARB	0.81	0.34-1.90	0.628
DOAC	0.51	0.23-1.12	0.095
Multivariable regression			
Loop diuretic	15.45	4.43-53.89	<0.001
MRA	1.54	0.81-2.95	0.192
Digoxin	1.54	0.88-2.67	0.128
Pulmonary vasodilator	1.39	0.79-2.45	0.247
Other antiarrhythmic	1.37	0.76-2.48	0.292
Other beta blocker	1.02	0.53-1.96	0.950
Anticoagulant	1.01	0.53-1.94	0.972

MRA = mineralocorticoid receptor; other abbreviations as in Table 3.

A multivariable Cox regression model was developed to evaluate the relationship between several medications that were independently associated with transplant-free survival (Table 4). In this model, loop diuretic prescription remained significant (HR: 15.45; *P* < 0.001). To account for patients establishing care at different ages, age at the first ACHD visit was incorporated into the models, which did not significantly change these results (Supplemental Table 4).

**Figure 1:**
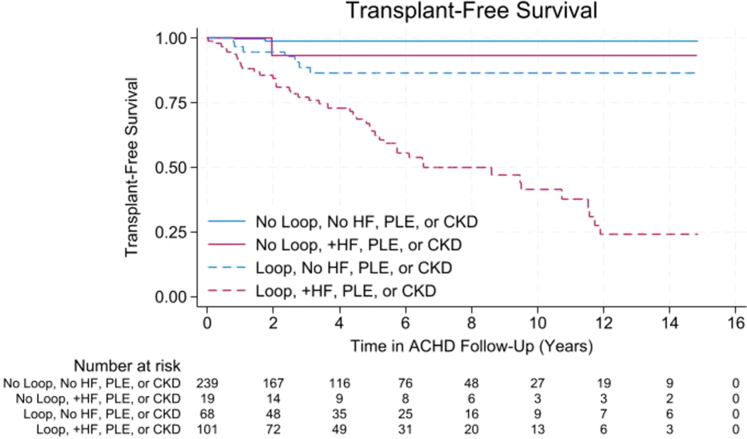
Transplant-Free Survival for Patients Prescribed Loop Diuretic Agents Transplant-free survival is decreased in patients prescribed loop diuretic agents (dashed lines) compared with those never prescribed loop diuretic agents (solid lines), even when accounting for presence of heart failure (HF), protein losing enteropathy (PLE), and chronic kidney disease (CKD). Log-rank test *P* < 0.001. ACHD = adult congenital heart disease.

To better account for patient characteristics and comorbidities associated with prescription of loop diuretic agents, sensitivity analysis was performed using logistic regression, incorporating clinically appropriate variables significant on univariable regression into the multivariable model (Supplemental Table 5). Factors significantly associated with prescription of loop diuretic agents on multivariable logistic regression were incorporated into the Cox regression multivariable model (Table 5). The association between loop diuretic agents and transplant-free survival remained significant (HR: 9.49; *P* < 0.001). Systolic and/or diastolic heart failure (HR: 2.63; *P* = 0.005) was also significant in the final loop diuretic agents model, whereas chronic kidney disease and protein losing enteropathy approached significance (Table 5). Survival curves were generated for patients prescribed loop diuretic agents with and without these comorbidities (Figure 1).

**Table 5:** Association Between Loop Diuretics and DOAC and Transplant-free Survival From First ACHD Encounter Incorporating Patient Characteristics and Comorbidities

	HR	95% CI	*P* Value
Loop diuretic agents	9.49	2.69-33.55	<0.001
Heart failure	2.63	1.33-5.18	0.005
Chronic kidney disease	1.77	0.94-3.31	0.076
PLE	1.72	0.89-3.34	0.109
Cirrhosis	1.54	0.75-3.19	0.239
Cyanosis	1.25	0.72-2.18	0.435
Age at first ACHD visit, y	1.009	0.979-1.040	0.579

PLE = protein losing enteropathy; other abbreviations as in Table 1.

### Medication burden and outcomes

**Figure 2:**
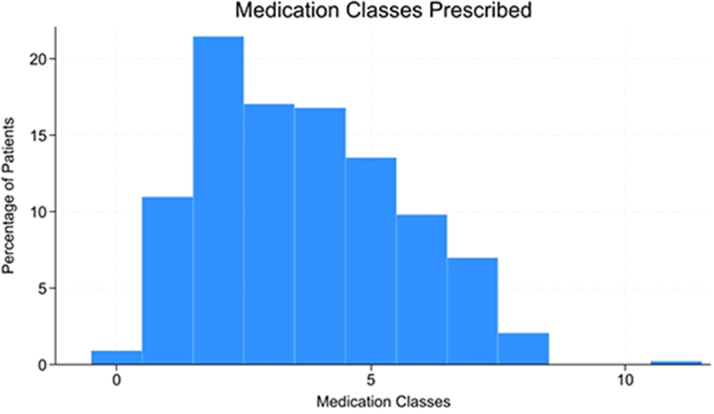
Number of Medication Classes Prescribed Histogram representing number of medication classes prescribed to adult patients with Fontan palliation. Most patients were prescribed 2 medication classes during the follow-up in ACHD clinic

The number of medication classes prescribed to patients throughout their follow-up in ACHD clinic is summarized in Figure 2. It was most common for patients to be prescribed 2 classes of medications.

On univariable analysis, there was an association between burden of medications and transplant-free survival (Table 6). Patients prescribed 3 to 4 (HR: 10.20; *P* = 0.026) or 5 or more medication classes had an increased hazard of transplant or death (HR: 33.22; *P* = 0.001).

**Table 6:** Association Between Medication Burden and Transplant-Free Survival From First ACHD Encounter

Medication Classes Prescribed	HR	95% CI	*P* Value
0-2	-	-	-
3-4	10.20	1.32-79.05	0.026
5 or more	33.22	4.57-242	0.001

Abbreviations as in Table 1.

The logistic regression sensitivity analysis was repeated to identify patient characteristics and comorbidities associated with medication burden (Supplemental Table 6). Factors found to be significantly associated with medication burden were included in a final multivariable Cox model (Table 7). Following adjustment for significant confounders, there was no association between increased mediation burden and transplant-free survival. However, the comorbidities of systolic and/or diastolic heart failure (HR: 6.02, *P* < 0.001) and cirrhosis (HR: 2.64, *P* = 0.014) were significant.

**Table 7:** Association Between Medication Classes Prescribed and Transplant-Free Survival Incorporating Patient Characteristics and Comorbidities

	HR	95% CI	*P* Value
Number of medications (reference = 0-2)			
3-4	7.45	0.92-60.53	0.060
5 or More	7.21	0.83-62.85	0.074
Race (reference = White)			
Asian	-		
Black	1.62	0.69-3.80	0.271
Unknown	2.13	0.74-6.14	0.161
Other	2.93	0.79-10.90	0.108
Diagnosis (reference = tricuspid atresia)			
HLHS	2.19	0.98-4.90	0.057
DORV	0.97	0.39-2.40	0.946
DILV	1.88	0.77-4.58	0.167
Unbalanced atrioventricular canal	1.51	0.60-3.78	0.379
PA/IVS	-		
Other	0.82	0.17-3.85	0.799
Arrhythmia	0.76	0.35-1.63	0.478
Heart failure	6.02	2.74-13.25	<0.001
Stroke/transient ischemic attack	1.11	0.60-2.04	0.750
Cirrhosis	2.64	1.21-5.76	0.014
Venous thromboembolism	1.49	0.75-2.95	0.256
Diabetes	1.51	0.51-4.46	0.459

Abbreviations as in Table 1.

## Discussion

This retrospective study reports one of the first large-scale studies of prescription patterns within the population of adults living with Fontan circulation at a single ACHD center (Central Illustration). We demonstrate a significant negative association between loop diuretic prescriptions and transplant-free survival. When accounting for patient comorbidities, no other medication class showed a significant association with transplant-free survival.

**Central Illustration:**
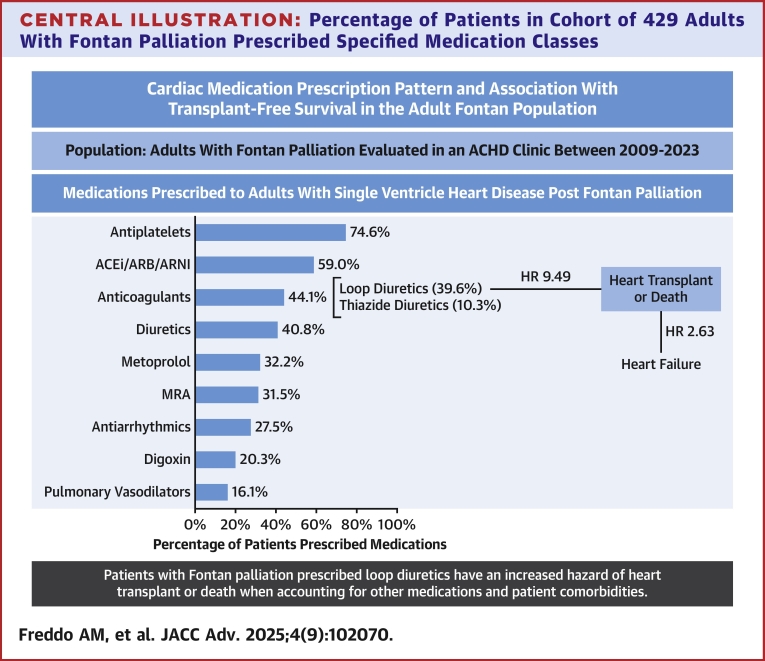
Percentage of Patients in Cohort of 429 Adults With Fontan Palliation Prescribed Specified Medication Classes Of medications studied, only patients prescribed loop diuretic agents have an increased hazard of heart transplant or death (HR: 9.49; *P* < 0.001) when accounting for other medications and patient comorbidities. In this model, heart failure also has a significant association with heart transplant or death. ACEi = angiotensin-converting enzyme inhibitor; ACHD = adult congenital heart disease; ARB = angiotensin receptor blocker; ARNI = angiotensin receptor/neprilysin inhibitor; MRA = mineralocorticoid receptor.

In the pediatric Fontan population, there are a limited number of reports describing medication use, including analysis of claims databases and larger single-center studies to determine prevalence of medication use. A large study of claims data from over 4,000 pediatric patients with hypoplastic left heart syndrome or tricuspid atresia^6^ demonstrated that patients were prescribed fewer medications than in our study; over half of the pediatric population were not prescribed any medications, excluding antiplatelet agents. In that study, the most prescribed medications were ACEi and ARB (38%), diuretic agents (15%) and pulmonary vasodilators (6%); older patients (13-19 years old) were even less likely to be prescribed these medication classes than younger patients.^6^ This result contrasts with our study, where over 40% of patients were prescribed diuretic agents and over 15% were prescribed pulmonary vasodilators. This is likely in large part due to differences in both overall patient morbidity in the pediatric vs adult population and prescriber practices between pediatric and adult congenital cardiologists.

In addition to the most frequently prescribed medications, it is important to highlight less frequently used medications in this population. Without established guidelines for adults living with congenital heart disease and heart failure, we find a low proportion of patients prescribed more novel heart failure therapies, such as angiotensin receptor/neprilysin inhibitor (4, 0.9%) and sodium-glucose cotransporter-2 inhibitors (8, 1.9%), mainstays of acquired heart failure treatment, despite just over 20% of our population having heart failure. It is possible that these low proportions of patients treated with these more novel therapies is center-specific, although there are limited data about their use in patients living with Fontan circulation, largely case series.^7^^,^^8^ Evaluating practices at multiple centers in a prospective fashion could build on this work and provide more insight into how best to use these medications in this patient population.

### Medications prescribed and outcomes

There are few studies examining the relationship between medication prescriptions and outcomes in adults with Fontan circulation. In this study, although a cause-and-effect relationship cannot be inferred between medication use and outcomes, the relationship between loop diuretic agents and transplant-free survival supports the concept of the Fontan circulation as one of chronic circulatory failure, which may include systolic or diastolic dysfunction as well as Fontan obstruction, increased pulmonary arterial pressures and/or pulmonary vascular resistance, and/or lymphatic complications.

Although older patients and those with comorbidities such as cirrhosis and protein losing enteropathy are more likely to be prescribed loop diuretic agents, these comorbidities alone do not explain this observed association. Patients who develop congestive symptoms, even without these comorbidities, are likely to be prescribed diuretic agents, which can improve symptoms.^9^ Uniquely, patients with Fontan failure have increased systemic congestion due to passive systemic venous return and pulmonary blood flow.^3^^,^^10^ Diuretic agents are used to alleviate venous congestion and the associated symptoms even in the absence of other comorbidities. This likely represents a sicker patient population with clinically worse prognosis. Conversely, those lacking these symptoms, and as a result who are not prescribed diuretic agents, likely represent a healthier cohort of patients.

We find that patients prescribed medications used to target other results of Fontan circulatory failure, including increased pulmonary vascular resistance (pulmonary vasodilators) and systolic dysfunction (MRA and beta blockers), have a significantly increased risk of transplant or death on univariable analysis. However, these associations do not hold when accounting for multiple medication classes. This suggests that the prescription of loop diuretic agents and presence of congestive symptoms are the most strongly associated with the sickest group of patients living with Fontan circulation when compared with other manifestations of Fontan circulatory failure.

Unlike all other medications studied, the only medication class that trended toward a decreased risk of transplant or death on univariable analysis was DOAC. Patients with Fontan circulation have an increased risk of thromboembolism that confers great morbidity and mortality.^11^, ^12^, ^13^ DOAC have been considered an option for anticoagulation in patients living with Fontan circulation more recently due to studies demonstrating their noninferiority compared with warfarin in adult patients living with Fontan circulation.^14^^,^^15^ However, there is still much debate on the efficacy of this class.^15^^,^^16^ Our findings may reflect the recent adoption of DOAC medications and the smaller number of patients prescribed them in our cohort. As their use increases and becomes more widespread, it will be important to see if this trend toward increased transplant-free survival persists or if it is attributable to the recent adoption of this medication.

### Medication burden and outcomes

We found that increased medication burden (regardless of class) was adversely associated with transplant-free survival on univariable analysis. However, patients who are prescribed more medications also have increased comorbidities, such as heart failure and cirrhosis (Supplemental Table 6), compared with those prescribed fewer medication classes. These comorbidities were significantly associated with transplant-free survival on multivariable analysis, whereas the number of medication classes prescribed was not. This emphasizes the importance of controlling for patient characteristics and comorbidities that may be associated with prescription practices.

### Study Limitations

This study is limited by being retrospective in nature. It also was performed at a single quaternary referral center, which may not be generalizable to other practice settings. Because it was performed in an adult-based setting, there is selection bias as only patients who survive to adulthood are included in the study. The justification for why specific patients were prescribed specific medication classes was not captured by this analysis, and medications may be prescribed for multiple indications. We are unable to assess patient adherence to prescribed medications. It also does not speak to what medications may improve patient outcomes. Although many clinical variables were captured, there is the potential for additional, unmeasured confounders. A significant association between medications prescribed and outcomes does not imply causality. Several models and associations were investigated without *P* value adjustment for multiple comparisons. For this reason, our findings, including CIs, should be interpreted with caution and serve as motivation for future refined questions. Nevertheless, this important work will inform future retrospective cohort studies, as well as prospective studies, in improving treatment for patients living with ACHD.

## Conclusions

This is one of the first large-scale studies of medications prescribed in adults living with Fontan circulation. The most common medications prescribed were antiplatelet agents, ACEi/ARB, anticoagulants, antiarrhythmics, and diuretic agents. Loop diuretic agents had the strongest association with transplant-free survival. Patients prescribed loop diuretic agents had significantly worse transplant-free survival, with and without adjustment for patient comorbidities.

Perspectives **COMPETENCY IN MEDICAL KNOWLEDGE:** Adult patients living with Fontan circulation are treated with a wide variety of cardiac medications; of these, loop diuretic agents has the strongest association with decreased transplant-free survival.

**TRANSLATIONAL OUTLOOK:** This study can inform future prospective studies to determine changes in laboratory or clinical testing after therapy initiation as well as form the basis for guideline-directed therapy for patients with Fontan circulation.

## Funding support and author disclosures

This study was funded by Matthew Hearts of Hope Foundation. This work was supported in part by the Cardiac Center Clinical Research Core at the Children’s Hospital of Philadelphia. The authors have reported that they have no relationships relevant to the contents of this paper to disclose.
