# Supplementary material for: Cardiac Medication Prescription Pattern and Association With Transplant-free Survival in the Adult Fontan Population
Source: JACC Adv. 2025 Aug 20;4(9):102070. doi: 10.1016/j.jacadv.2025.102070 (PMC12539492; doi:10.1016/j.jacadv.2025.102070)
Supplement: Supplementary data [file mmc1.docx]

Supplemental Table 1: ICD9/10 codes utilized for patient identification.

| **Diagnosis Name** | **ICD9 Code** | **ICD10 Code** |
| --- | --- | --- |
| Status post Fontan procedure | V45.89 | Z98.890 |
| S/P Fontan procedure | V45.89 | Z98.890 |
| Post-Fontan protein-losing enteropathy | 579.8 | K90.49, Z98.890 |
| Status post Fontan operation | V45.89 | Z98.890 |
| Status post hemi-Fontan operation | V45.89 | Z98.890 |
| S/P hemi-Fontan operation | V45.89 | Z98.890 |

Supplemental Table 2: Medication Classifications

| Medication Super-Class | Medication Class | Medication (generic) |
| --- | --- | --- |
| ACEi/ARB/ARNI | ACEi | captopril |
|  |  | enalapril |
|  |  | lisinopril |
|  |  | quinapril |
|  |  | ramipril |
|  | ARB | irbesartan |
|  |  | losartan |
|  |  | olmesartan |
|  |  | telmisartan |
|  |  | valsartan |
|  | ARNI | sacubitril-valsartan |
| Anticoagulants | DOAC | apixaban |
|  |  | dabigatran |
|  |  | edoxaban |
|  |  | rivaroxaban |
|  | Heparin Analog | dalteparin |
|  |  | enoxaparin |
|  |  | heparin |
|  | Warfarin | warfarin |
| Diuretics | Loop Diuretic | bumetanide |
|  |  | furosemide |
|  |  | torsemide |
|  | Thiazide Diuretic | chlorothiazide |
|  |  | chlorthalidone |
|  |  | HCTZ |
|  |  | indapamide |
|  |  | metolazone |
|  | Other Diuretic | amiloride |
| Anti-Arrhythmics | Other Anti-Arrhythmic | amiodarone |
|  |  | dofetilide |
|  |  | flecainide |
|  |  | mexiletine |
|  |  | propafenone |
|  |  | sotalol |
|  | Anti-Arrhythmic Beta Blocker | atenolol |
|  |  | nadolol |
|  |  | propranolol |
|  | Anti-Arrhythmic Calcium Channel Blocker | diltiazem |
|  |  | verapamil |
| Antiplatelets | | aspirin |
|  |  | clopidogrel |
|  |  | ticagrelor |
| Metoprolol | | metoprolol succinate |
|  |  | metoprolol tartrate |
| Mineralocorticoid Receptor Antagonists | | eplerenone |
|  |  | spironolactone |
| Digoxin | | digoxin |
| Pulmonary Vasodilators | | bosentan |
|  |  | macitentan |
|  |  | sildenafil |
|  |  | tadalafil |
|  |  | treprostinil |
|  |  | udenafil |
| Other Beta Blockers | | bisoprolol |
|  |  | carvedilol |
|  |  | labetalol |
|  |  | pindolol |
| Statins | | atorvastatin |
|  |  | lovastatin |
|  |  | rosuvastatin |
|  |  | simvastatin |
| GLP1 | | dulaglutide |
|  |  | liraglutide |
|  |  | semaglutide |
| Other Calcium Channel Blockers | | amlodipine |
|  |  | felodipine |
| SGLT2i | | dapagliflozin |
|  |  | empagliflozin |

Supplemental Table 3: Extra-Cardiac Comorbidities.

| **Category** | **N (%)** |
| --- | --- |
| Gastrointestinal Comorbidities |  |
| Focal Liver Lesions | 99 (23.1) |
| Hepatocellular Carcinoma | 10 (10.1) |
| Not Hepatocellular Carcinoma | 25 (25.3) |
| Indeterminate | 64 (64.6) |
| Cirrhosis | 184 (42.9) |
| Renal Comorbidities |  |
| Chronic Kidney Disease | 30 (7.0) |
| Neurologic Comorbidities |  |
| Migraine | 68 (15.9) |
| Seizure Disorder | 39 (9.1) |
| Hematologic-Oncologic Comorbidities |  |
| Venous Thromboembolism | 54 (12.6) |
| Bleeding Disorder | 22 (5.1) |
| Arterial Thrombus | 16 (3.7) |
| Hypercoagulable State | 6 (1.4) |
| Psychiatric Comorbidities |  |
| Anxiety | 114 (26.6) |
| Depression | 84 (19.6) |
| Endocrine Comorbidities |  |
| Diabetes | 15 (3.5) |
| Thyroid Disease | 62 (14.5) |
| Obesity | 65 (15.2) |
| Genetic Syndrome | 7 (1.6%) |

Supplemental Table 4: Association Between Medication Class Prescribed and Transplant-Free Survival from First ACHD Encounter Including Age at First ACHD Encounter

| Single Medication Class Regressions | | | |
| --- | --- | --- | --- |
| Super-Classes | | | |
| MRA^a^ | 4.79 | 2.59 – 8.88 | <0.001 |
| Pulmonary Vasodilator^a^ | 2.70 | 1.55 – 4.72 | <0.001 |
| Other Beta Blockers^a^ | 2.15 | 1.15 – 4.01 | 0.017 |
| Anticoagulant^a^ | 1.98 | 1.06 – 3.68 | 0.032 |
| Digoxin^a^ | 1.86 | 1.08 – 3.22 | 0.026 |
| Anti-Arrhythmic^a^ | 1.66 | 0.93 – 2.97 | 0.088 |
| Metoprolol^a^ | 1.13 | 0.66 – 1.94 | 0.660 |
| ACEi/ARB/ARNI^a^ | 0.87 | 0.51 – 1.51 | 0.626 |
| Classes | | | |
| Loop Diuretic | 22.73 | 6.99 – 73.96 | <0.001 |
| Warfarin^a^ | 2.59 | 1.45 – 4.65 | 0.001 |
| Other Anti-Arrhythmic^a^ | 2.17 | 1.23 – 3.83 | 0.007 |
| ACEi^a^ | 0.99 | 0.58 – 1.71 | 0.985 |
| Anti-Arrhythmic Beta Blockers^a^ | 0.83 | 0.37 – 1.84 | 0.649 |
| ARB^a^ | 0.58 | 0.24 – 1.40 | 0.228 |
| DOAC^a^ | 0.48 | 0.22 – 1.07 | 0.071 |
| Multivariable Regression^a^ | | | |
| Loop Diuretic | 14.68 | 4.17 – 51.66 | <0.001 |
| MRA | 1.54 | 0.81 – 2.94 | 0.192 |
| Digoxin | 1.48 | 0.85 – 2.60 | 0.169 |
| Pulmonary Vasodilator | 1.37 | 0.78 – 2.41 | 0.270 |
| Other Anti-Arrhythmic | 1.30 | 0.71 – 2.38 | 0.393 |
| Other Beta Blocker | 1.07 | 0.55 – 2.07 | 0.852 |
| Anticoagulant | 1.01 | 0.53 – 1.93 | 0.976 |

a. Age at first ACHD encounter was significant in these models.

Supplemental Table 5: Association Between Patient Characteristics and Comorbidities and Loop Diuretic.

|  | **Univariable** | | | **Multivariable** | | |
| --- | --- | --- | --- | --- | --- | --- |
| **Characteristic** | **Odds Ratio** | **95% CI** | **p-Value** | **Odds Ratio** | **95% CI** | **p-Value** |
| Patient Characteristics | | | | | | |
| Male Gender | 0.96 | 0.65 – 1.42 | 0.856 | - | - | - |
| Age at First ACHD Visit, years | 1.168 | 1.121 – 1.216 | <0.001 | 1.157 | 1.079 – 1.241 | <0.001 |
| Race (reference = White) | | | | | | |
| Asian | - | - | - | - | - | - |
| Black | 1.56 | 0.83 – 2.91 | 0.167 | - | - | - |
| Unknown | 0.85 | 0.44 – 1.64 | 0.627 | - | - | - |
| Other | 0.74 | 0.27 – 2.03 | 0.564 | - | - | - |
| Hispanic/Latino Ethnicity | 0.49 | 0.18 – 1.38 | 0.179 | - | - | - |
| Primary Cardiac Diagnosis (reference = tricuspid atresia) | | | | | | |
| HLHS | 0.69 | 0.40 – 1.18 | 0.177 | - | - | - |
| DORV | 0.82 | 0.43 – 1.55 | 0.535 | - | - | - |
| DILV | 0.73 | 0.38 – 1.39 | 0.337 | - | - | - |
| Unbalanced AV Canal | 0.81 | 0.40 – 1.66 | 0.567 | - | - | - |
| PA/IVS | 0.38 | 0.14 – 1.01 | 0.053 | - | - | - |
| Other | 0.83 | 0.33 – 2.08 | 0.685 | - | - | - |
| Ventricular Morphology (reference = single left ventricle) | | | | | | |
| Single Right Ventricle | 0.96 | 0.65 – 1.44 | 0.859 | - | - | - |
| Mixed Ventricular Morphology | 1.06 | 0.48 – 2.34 | 0.879 | - | - | - |
| Heterotaxy | 0.95 | 0.52 – 1.72 | 0.855 | - | - | - |
| Fontan Type (reference = extracardiac) | | | | | | |
| Lateral Tunnel | 1.75 | 1.01 – 3.05 | 0.047 | 1.32 | 0.60 – 2.88 | 0.489 |
| Aortopulmonary | 7.33 | 3.50 – 15.35 | <0.001 | 0.67 | 0.19 – 2.34 | 0.530 |
| Other | 4.19 | 1.71 – 10.26 | 0.002 | 2.51 | 0.68 – 9.25 | 0.167 |
| Fenestration | 0.33 | 0.22 – 0.50 | <0.001 | 0.91 | 0.46 – 1.79 | 0.789 |
| Age at Fontan (months) | 1.013 | 1.009 – 1.018 | <0.001 | 0.997 | 0.990 – 1.005 | 0.480 |
| Fontan Revision | 4.24 | 2.31 – 7.78 | <0.001 | 1.35 | 0.53 – 3.46 | 0.533 |
| Follow-Up Time, Years | 1.00 | 1.00 – 1.00 | 0.762 | - | - | - |
| Selected Comorbidities | | | | | | |
| Arrhythmia | 3.17 | 2.07 – 4.85 | <0.001 | 0.94 | 0.48 – 1.81 | 0.843 |
| Pacemaker/ICD | 3.20 | 2.03 – 5.03 | <0.001 | 1.90 | 0.88 – 4.10 | 0.101 |
| Heart Failure | 16.82 | 8.92 – 31.71 | <0.001 | 8.49 | 3.92 – 18.37 | <0.001 |
| Pulmonary Hypertension | 23.15 | 3.02 – 177.80 | 0.003 | 6.46 | 0.61 – 67.97 | 0.120 |
| Cirrhosis | 6.03 | 3.94 – 9.23 | <0.001 | 2.81 | 1.60 – 4.94 | <0.001 |
| Chronic Kidney Disease | 11.51 | 3.94 – 33.63 | <0.001 | 4.71 | 1.10 – 20.15 | 0.037 |
| Thyroid Disease | 3.05 | 1.75 – 5.34 | <0.001 | 1.19 | 0.49 – 2.89 | 0.699 |
| Cyanosis | 3.18 | 2.03 – 4.97 | <0.001 | 3.32 | 1.15 – 9.54 | 0.026 |
| PLE | 25.34 | 5.95 – 107.92 | <0.001 | 45.17 | 7.58 – 269 | <0.001 |
| No Fontan Comorbidities | 0.27 | 0.18 – 0.40 | <0.001 | 1.81 | 0.68 – 4.86 | 0.238 |

Supplemental Table 6: Association Between Patient Characteristics and Comorbidities and Number of Medication Classes Prescribed.

|  | **Univariable** | | | **Multivariable** | | |
| --- | --- | --- | --- | --- | --- | --- |
| **Characteristic** | **Odds Ratio** | **95% CI** | **p-Value** | **Odds ratio** | **95% CI** | **p-Value** |
| 3 to 4 Medication Classes |  |  |  |  |  |  |
| Patient Characteristics | | | | | | |
| Male Gender | 1.13 | 0.71 – 1.80 | 0.599 | - | - | - |
| Age at First ACHD Visit, years | 1.13 | 1.07 – 1.19 | <0.001 | 1.10 | 1.02 – 1.19 | 0.020 |
| Race (reference = White) | | | | | | |
| Asian | 0.10 | 0.01 – 0.82 | 0.032 | 0.08 | 0.01 – 0.90 | 0.041 |
| Black | 0.63 | 0.28 – 1.42 | 0.267 | 0.82 | 0.31 – 2.17 | 0.689 |
| Unknown | 1.11 | 0.52 – 2.38 | 0.780 | 1.13 | 0.44 – 2.87 | 0.799 |
| Other | 1.28 | 0.39 – 4.18 | 0.677 | 2.01 | 0.50 – 8.01 | 0.324 |
| Hispanic/Latino Ethnicity | 0.65 | 0.22 – 1.87 | 0.421 | - | - | - |
| Primary Cardiac Diagnosis (reference = tricuspid atresia) | | | | | | |
| HLHS | 1.12 | 0.59 – 2.10 | 0.744 | 1.25 | 0.57 – 2.78 | 0.577 |
| DORV | 1.45 | 0.64 – 5.93 | 0.373 | 1.11 | 0.40 – 3.08 | 0.839 |
| DILV | 0.73 | 0.34 – 1.58 | 0.433 | 0.83 | 0.33 – 2.12 | 0.699 |
| Unbalanced AV Canal | 2.72 | 1.06 – 6.89 | 0.037 | 2.83 | 0.65 – 12.43 | 0.167 |
| PA/IVS | 0.69 | 0.24 – 1.97 | 0.488 | 1.10 | 0.31 – 3.88 | 0.880 |
| Other | 0.81 | 0.26 – 2.56 | 0.724 | 1.07 | 0.26 – 4.50 | 0.922 |
| Ventricular Morphology (reference = single left ventricle) | | | | | | |
| Single Right Ventricle | 1.49 | 0.92 – 2.41 | 0.099 | - | - | - |
| Mixed Ventricular Morphology | 1.20 | 0.44 – 3.19 | 0.723 | - | - | - |
| Heterotaxy | 2.53 | 1.15 – 5.53 | 0.020 | 0.78 | 0.20 – 3.07 | 0.725 |
| Fontan Type (reference = extracardiac) | | | | | | |
| Lateral Tunnel | 1.34 | 0.77 – 2.32 | 0.307 | 0.92 | 0.45 – 1.88 | 0.823 |
| Aortopulmonary | 2.59 | 0.93 – 7.17 | 0.069 | 0.62 | 0.13 – 2.91 | 0.547 |
| Other | 1.28 | 0.44 – 3.82 | 0.646 | 0.38 | 0.09 – 1.66 | 0.199 |
| Fenestration | 0.64 | 0.40 – 1.04 | 0.075 | 1.06 | 0.54 – 2.09 | 0.861 |
| Age at Fontan (months) | 1.01 | 1.01 – 1.02 | 0.001 | 1.01 | 1.00 – 1.02 | 0.176 |
| Fontan Revision | 1.35 | 0.55 – 3.32 | 0.508 | 0.51 | 0.15 – 1.75 | 0.287 |
| Follow-Up Time, Years | 1.00 | 1.00 – 1.00 | 0.371 | 1.00 | 1.00 – 1.00 | 0.936 |
| Comorbidities | | | | | | |
| Arrhythmia | 3.47 | 2.14 – 5.64 | <0.001 | 2.83 | 1.46 – 5.49 | 0.002 |
| Pacemaker/ICD | 1.69 | 0.91 – 3.12 | 0.097 | 1.22 | 0.52 – 2.88 | 0.649 |
| Heart Failure | 2.27 | 0.84 – 6.11 | 0.108 | 1.09 | 0.32 – 3.67 | 0.894 |
| Valve Repair | 0.59 | 0.14 – 2.51 | 0.472 | - | - | - |
| Valve Replacement | - | - | - | - | - | - |
| Pulmonary Hypertension | - | - | - | - | - | - |
| Endocarditis | 4.06 | 0.45 – 36.60 | 0.213 | - | - | - |
| Stroke/Transient Ischemic Attack | 2.97 | 1.49 – 5.93 | 0.002 | 2.84 | 1.25 – 6.48 | 0.013 |
| Cirrhosis | 3.56 | 2.08 – 6.11 | <0.001 | 2.89 | 1.51 – 5.56 | 0.001 |
| Focal Liver Lesions | 0.95 | 0.54 – 1.67 | 0.861 | - | - | - |
| Chronic Kidney Disease | 2.39 | 0.61 – 9.39 | 0.215 | 1.44 | 0.20 – 10.26 | 0.716 |
| Migraine | 1.04 | 0.56 – 1.95 | 0.894 | - | - | - |
| Seizure Disorder | 1.72 | 0.78 – 3.78 | 0.181 | - | - | - |
| Venous Thromboembolism | 1.26 | 0.48 – 3.29 | 0.638 | 0.92 | 0.29 – 2.88 | 0.885 |
| Bleeding Disorder | 0.76 | 0.28 – 2.10 | 0.598 | - | - | - |
| Hypercoagulable State | 1.99 | 0.18 – 22.20 | 0.573 | - | - | - |
| Arterial Thrombus | 2.53 | 0.48 – 13.33 | 0.271 | 3.20 | 0.51 – 20.13 | 0.215 |
| Anxiety | 0.99 | 0.58 – 1.70 | 0.973 | - | - | - |
| Depression | 1.36 | 0.76 – 2.46 | 0.309 | - | - | - |
| Diabetes | 3.03 | 0.31 – 29.37 | 0.341 | 10.46 | 0.62 – 177 | 0.104 |
| Thyroid Disease | 3.19 | 1.38 – 7.46 | 0.007 | 1.56 | 0.56 – 4.35 | 0.393 |
| Cyanosis | 1.93 | 1.06 – 3.53 | 0.030 | 0.62 | 0.15 – 2.48 | 0.496 |
| Fontan Thrombus | 3.16 | 0.99 – 10.07 | 0.051 | 1.34 | 0.25 – 7.34 | 0.734 |
| Pulmonary Arteriovenous Malformations | 2.05 | 0.68 – 6.17 | 0.198 | 0.75 | 0.17 – 3.25 | 0.702 |
| PLE | - | - | - | - | - | - |
| No Fontan Comorbidities | 0.41 | 0.24 – 0.69 | 0.001 | 0.35 | 0.08 – 1.41 | 0.139 |
| 5 or More Medication Classes | | | | | | |
| Patient Characteristics | | | | | | |
| Male Gender | 1.30 | 0.81 – 2.08 | 0.272 | - | - | - |
| Age at First ACHD Visit, years | 1.21 | 1.15 – 1.28 | <0.001 | 1.16 | 1.05 – 1.28 | 0.003 |
| Race (reference = White) | | | | | | |
| Asian | 0.11 | 0.01 – 0.88 | 0.037 | 0.37 | 0.03 – 4.52 | 0.436 |
| Black | 1.10 | 0.53 – 2.28 | 0.792 | 1.18 | 0.36 – 3.86 | 0.786 |
| Unknown | 0.91 | 0.41 – 2.03 | 0.819 | 2.33 | 0.74 – 7.38 | 0.151 |
| Other | 1.18 | 0.35 – 3.98 | 0.794 | 3.71 | 0.62 – 22.07 | 0.150 |
| Hispanic/Latino Ethnicity | 0.56 | 0.18 – 1.70 | 0.303 | - | - | - |
| Primary Cardiac Diagnosis (reference = tricuspid atresia) | | | | | | |
| HLHS | 0.84 | 0.44 – 1.60 | 0.602 | 3.03 | 1.04 – 8.83 | 0.042 |
| DORV | 1.60 | 0.73 – 3.49 | 0.239 | 2.69 | 0.76 – 9.54 | 0.126 |
| DILV | 0.64 | 0.30 – 1.38 | 0.255 | 1.16 | 0.32 – 4.26 | 0.820 |
| Unbalanced AV Canal | 1.75 | 0.66 – 4.66 | 0.261 | 4.24 | 0.68 – 26.47 | 0.122 |
| PA/IVS | 0.64 | 0.22 – 1.80 | 0.397 | 1.41 | 0.24 – 8.26 | 0.702 |
| Other | - | - | - | 2.53 | 0.41 – 15.77 | 0.319 |
| Ventricular Morphology (reference = single left ventricle) | | | | | | |
| Single Right Ventricle | 1.39 | 0.85 – 2.27 | 0.186 | - | - | - |
| Mixed Ventricular Morphology | 1.48 | 0.58 – 3.78 | 0.412 | - | - | - |
| Heterotaxy | 2.10 | 0.94 – 4.71 | 0.070 | 0.46 | 0.10 – 2.22 | 0.334 |
| Fontan Type (reference = extracardiac) | | | | | | |
| Lateral Tunnel | 2.05 | 1.06 – 3.94 | 0.032 | 0.83 | 0.31 – 2.23 | 0.707 |
| Aortopulmonary | 13.87 | 5.16 – 37.71 | <0.001 | 0.85 | 0.15 – 4.91 | 0.858 |
| Other | 3.74 | 1.30 – 10.91 | 0.015 | 0.59 | 0.10 – 3.48 | 0.558 |
| Fenestration | 0.36 | 0.22 – 0.59 | <0.001 | 1.69 | 0.70 – 4.05 | 0.242 |
| Age at Fontan (months) | 1.02 | 1.01 – 1.03 | <0.001 | 1.01 | 0.99 – 1.02 | 0.339 |
| Fontan Revision | 5.00 | 2.29 – 10.91 | <0.001 | 1.05 | 0.28 – 3.93 | 0.941 |
| Follow-Up Time, Years | 1.00 | 1.00 – 1.00 | <0.001 | 1.00 | 1.00 – 1.00 | 0.077 |
| Comorbidities | | | | | | |
| Arrhythmia | 9.20 | 5.29 – 16.01 | <0.001 | 7.42 | 3.05 – 18.03 | <0.001 |
| Pacemaker/ICD | 4.13 | 2.31 – 7.39 | <0.001 | 1.23 | 0.44 – 3.38 | 0.695 |
| Heart Failure | 25.79 | 10.70 – 62.18 | <0.001 | 9.09 | 2.78 – 29.73 | <0.001 |
| Valve Repair | 2.36 | 0.80 – 7.03 | 0.119 | - | - | - |
| Valve Replacement^a^ | 1.07 | 0.35 – 3.03 | 0.947 | - | - | - |
| Pulmonary Hypertension^a^ | 4.44 | 1.22 – 16.12 | 0.023 | - | - | - |
| Endocarditis | 7.54 | 0.91 – 62.18 | 0.061 | - | - | - |
| Stroke/Transient Ischemic Attack | 3.90 | 1.97 – 7.69 | <0.001 | 2.19 | 0.81 – 5.92 | 0.124 |
| Cirrhosis | 10.70 | 6.11 – 18.73 | <0.001 | 5.71 | 2.63 – 12.39 | <0.001 |
| Focal Liver Lesions | 1.36 | 0.79 – 2.34 | 0.271 | - | - | - |
| Chronic Kidney Disease | 7.85 | 2.27 – 27.11 | 0.001 | 1.42 | 0.18 – 11.29 | 0.742 |
| Migraine | 0.93 | 0.49 – 1.77 | 0.821 | - | - | - |
| Seizure Disorder | 0.93 | 0.38 – 2.27 | 0.873 | - | - | - |
| Venous Thromboembolism | 5.87 | 2.61 – 13.20 | <0.001 | 5.24 | 1.55 – 17.74 | 0.008 |
| Bleeding Disorder | 0.67 | 0.23 – 1.93 | 0.462 | - | - | - |
| Arterial Thrombus | 4.85 | 1.03 – 23.10 | 0.045 | 6.59 | 0.86 – 50.30 | 0.069 |
| Hypercoagulable State | 3.13 | 0.32 – 30.57 | 0.325 | - | - | - |
| Anxiety | 1.43 | 0.84 – 2.41 | 0.181 | - | - | - |
| Depression | 1.31 | 0.72 – 2.39 | 0.382 | - | - | - |
| Diabetes | 12.18 | 1.55 – 95.58 | 0.017 | 29.76 | 1.61 – 548 | 0.022 |
| Thyroid Disease | 4.85 | 2.14 – 10.91 | <0.001 | 0.89 | 0.26 – 2.98 | 0.847 |
| Cyanosis | 3.67 | 2.08 – 6.55 | <0.001 | 0.87 | 0.19 – 4.06 | 0.859 |
| Fontan Thrombus | 8.76 | 2.97 – 25.79 | <0.001 | 2.03 | 0.33 – 12.38 | 0.443 |
| Pulmonary Arteriovenous Malformations | 4.62 | 1.68 – 12.68 | 0.003 | 1.75 | 0.36 – 8.48 | 0.488 |
| PLE^a^ | 1.63 | 0.76 – 3.53 | 0.211 | - | - | - |
| No Fontan Comorbidities | 0.71 | 0.10 – 0.28 | <0.001 | 0.35 | 0.07 – 1.69 | 0.190 |

a. Univariable analyses for these characteristics done using the reference group as those patients prescribed 3 or 4 medication classes, as there were zero patients with these characteristics prescribed 0 to 2 medications. The reference group for the remaining characteristics is the group of patients prescribed 0 to 2 medication classes.
